# Partial LRR over complete LRR: a preferable option - an FEA study of A - B trochlear dysplasia with critical abnormalities of patella alta and TT – TG value

**DOI:** 10.3389/fbioe.2025.1473110

**Published:** 2025-04-17

**Authors:** Hanyu Wang, Elena Tahir, Huida Wang, Zhi Zhang, Xing Ma

**Affiliations:** Department of Orthopedics, The First Affiliated Hospital of Xi’an Jiao Tong University, Xi’an, China

**Keywords:** patellar instability (PI), patellofemoral joint (PFJ), lateral retinacular release (LRR), medial patellofemoral ligament (MPFL), finite element analysis (FEA)

## Abstract

**Objective:**

This study analyzed the effects of medial patellofemoral ligament (MPFL) injury and varying degrees of lateral retinacular release (LRR) on patellofemoral joint (PFJ) contact pressure using finite element analysis (FEA).

**Methods:**

A PFJ FE model was developed and validated at four knee flexion angles (0°, 30°, 60°, and 90°) using imaging data from a patient with A-B trochlear dysplasia and critical abnormalities of patella alta and tubercle-trochlear groove (TT-TG) value. MPFL injury was simulated by inhibiting its function, while LRR was modeled by adjusting the stiffness of the lateral retinaculum. Changes in PFJ contact pressure were systematically analyzed.

**Results:**

At 0° flexion, LRR led to increased PFJ pressure with an intact MPFL, whereas it resulted in a reduction with a ruptured MPFL. At 30° flexion, partial LRR didn’t elevate PFJ pressure when MPFL was intact, while complete LRR did with both intact and ruptured MPFL. At 60° flexion, partial LRR effectively reduced PFJ pressure, but complete release reversed this effect. At 90° flexion, PFJ pressure increased with the extent of LRR, irrespective of MPFL integrity. Specifically, complete LRR led to an increase in medial pressure, resulting in a shift of the pressure center from lateral to medial at 30° and 60° flexion.

**Clinical Implications:**

This study provides new theoretical basis for the expected outcomes of varying degrees of LRR, which helps clinicians better conduct preoperative planning, especially in avoiding over - aggressive LRR procedures which may not yield improved outcomes.

**Conclusion:**

In patients with A- B trochlear dysplasia and critical abnormalities, excessive LRR does not consistently lower PFJ pressure but rather increases medial compartment pressure, suggesting that partial release may be a more effective and precise surgical approach in these patients.

## 1 Introduction

Patellar instability is frequently associated with multiple anatomical risk factors, especially in patients experiencing recurrent patellar dislocation. More than half (58.3%) of these patients present with two or more concurrent risk factors, with patellar alta and a high tubercle-trochlear groove (TT-TG) value observed in 60% and 41.7% of cases, respectively (Huntington et al., 2020; Steensen et al., 2015). Treatment protocols are well-established for cases of patellar instability attributable to trauma or severe skeletal deformities. Traumatic instability is typically managed through medial patellofemoral ligament (MPFL) reconstruction, while severe deformities often require corrective surgery targeting specific risk factors, alongside MPFL reconstruction. For example, trochleoplasty is indicated for severe trochlear dysplasia (types C-D), and tibial tuberosity osteotomy and transposition are preferred for patients with marked patellar alta and elevated TT-TG values (Lamplot et al., 2022).

However, a significant void exists in the existing literature regarding patients who have multiple anatomical risk factors, especially critical yet less severe anatomical abnormalities. Current research has inadequately explored the unique biomechanics of such pathological patellofemoral joints, let alone described the outcomes of treating this patient subset with different soft tissue surgeries. Therefore, the optimal surgical approach in the treatment of this important clinical scenario is still disputed.

In the state of knee flexion from 0° to 30°, the patella demonstrates maximal range of motion relative to the femur, with the MPFL providing critical stabilizing support. Clinically, it represents a phase with an elevated risk of lateral patellar dislocation, making positive results in the patellar apprehension test indicative of instability. Moreover, lateral displacement of the patella beyond three-quarters of its width indicates compromised medial stabilizing structures, often associated with weakness or damage. The range of 0°–60° of flexion corresponds to angles typically observed during ambulation and moderate exertional activities, such as walking and running, making it essential for assessing dynamic stability of the PFJ (Kumbhalkar et al., 2021). Analyzing patellar mechanics within this flexion range provides critical insights into joint behavior under dynamic loading conditions common to daily activities. At 90° flexion, the knee achieves a position comparable to functional scenarios, such as ascending stairs or transitioning to a standing position from a seated position (Kumbhalkar et al., 2021). These flexion angles are vital for understanding key phases of patellar mechanics and offer deeper insights into joint stability and biomechanical behavior across various functional conditions.

Given the biomechanical complexity of patellar instability, various surgical techniques have been explored to address these risk factors. Among these, lateral retinacular release (LRR) is often performed in combination with MPFL reconstruction, especially in cases with lateral patellar instability. Initially developed to address excessive lateral pressure syndrome (ELPS), LRR has shown significant potential to reduce lateral patellofemoral pressure, improve patellar tilt, and lower lateral patellofemoral impingement in patients unresponsive to conservative treatments (Wang et al., 2021a; Huddleston et al., 2023; Kamalapathy et al., 2022).

Despite its therapeutic benefits, the use of LRR is not without risks. Excessive lateral release can lead to complications, including medial patellar instability, pain transferred to the medial patellofemoral area, reduced muscle strength, and even quadriceps muscle atrophy (Huddleston et al., 2023; Douiri et al., 2022). These issues can disrupt the patellofemoral forces essential for stable knee motion across various flexion and extension angles, thereby compromising joint stability (Kumbhalkar et al., 2021). While FEA is widely applied in studying bone and joint biomechanics (Kanu et al., 2021; Kanu et al., 2020), relatively fewer studies have specifically examined patellofemoral joint (PFJ) contact pressure, leaving gaps in understanding this key aspect. A study by Kheir et al., which utilized a finite element (FE) model of the patellofemoral joint (PFJ), found that combining MPFL reconstruction with LRR reduced PFJ contact pressure and area by approximately 40% compared to both the intact MPFL model and MPFL reconstruction alone (Kheir et al., 2022). However, their study also noted a 20% increase in lateral patellar displacement with knee flexion, suggesting a heightened risk of patellar instability following LRR. These findings align with those of Cancienne et al., who conducted biomechanical testing on cadaveric models, showing significantly greater lateral displacement in the LRR group, increasing nearly 30% at 0° knee flexion, with variations ranging from 6% to 9% across angles of 10°–90°(Cancienne et al., 2019). These results highlight the potential risks associated with LRR, particularly when combined with other procedures.

Furthermore, the ongoing debate regarding the routine combination of MPFL reconstruction with LRR for recurrent patellar dislocation reflects a broader uncertainty in the field. A prospective study by Wang et al. reported that patients undergoing the combined procedure showed better patient-reported outcome measures (PROMs) than those who received MPFL reconstruction alone; however, there were no significant differences in stability-related metrics, such as congruence angle (CA), patellar tilt angle (PTA), and lateral patellofemoral angle (LPFA) (Wang et al., 2021b). In contrast, a meta-analysis by Migliorini et al. found no statistically significant improvements between the combined and standalone procedures in terms of re-dislocation rates, positive fear tests, range of motion (ROM), complications, or revision surgeries, indicating insufficient evidence to support the routine use of LRR alongside MPFL reconstruction (Migliorini et al., 2020).

There may be the following reasons for the inconsistent conclusions in prior research. Firstly, due to ethical constraints in clinical studies, there often exist confounding factors from combined procedures, which makes it difficult to isolate the pure effect of lateral retinacular release (LRR). Secondly, the inconsistent definition of patient types across different studies has led to discrepant research conclusions. Different criteria for patient selection have made it challenging to compare and generalize the results. Thirdly, existing research has largely focused on whether to perform LRR, without exploring the impact of varying degrees of lateral release. The degree of LRR can significantly influence the biomechanical outcomes, but this aspect has been overlooked. Finally, differences in surgical techniques such as arthroscopic *versus* open release and variations in incision size, may also lead to conflicting outcomes and make it difficult to draw definitive conclusions about the biomechanical effects of LRR (Hamawandi et al., 2022).

Thus, the primary aim of this study was to investigate the biomechanical effects of MPFL injury and varying degrees of LRR on PFJ contact pressures using an FE model of the knee joint characterized by mild trochlear dysplasia, patellar alta, and an elevated TT-TG value (type A-B femoral trochlear dysplasia, Insall-Salvati (I-S) ratio ≈1.4, TT-TG value ≈20 mm). We hypothesized that MPFL rupture would lead to an increase in PFJ pressure at specific knee flexion angles and that partial LRR (50%) would alter PFJ pressure; however, extending the release beyond this threshold would not consistently result in a decrease in PFJ pressure.

## 2 Methods

### 2.1 Model construction

This was a retrospective finite element analysis study, which underwent review and received approval from The Ethics Committee of the First Affiliated Hospital of Xi’an Jiao Tong University. High-resolution computed tomography (CT) and magnetic resonance (MR) images of the knee joint were acquired from a 19-year-old male patient characterized by the following aspects: 1. Skeletal maturity, or closed epiphysis. 2. More than 2 episodes of dislocation. 3. Positive patellar apprehension test and J - sign. 4. Imaging - indicated medial patellofemoral ligament (MPFL) rupture. 5. Multiple anatomical risk factors, especially critical yet less severe anatomical abnormalities (type A-B by Dejour’s classification femoral trochlear dysplasia, Insall-Salvati (I-S) ratio ≈1.4, TT-TG value ≈20 mm). Morphological parameters, including the tibial tuberosity-trochlear groove (TT-TG) distance, Insall-Salvati (I-S) ratio, and patellar tilt angle, were measured for this patient and are provided in Table 1.

**TABLE 1:** Patient information.

Parameter	Value
Gender	Male
Age (years)	19
Tubercle-trochlear groove (TT-TG) value (mm)	21.8
Insall-Salvati ratio	1.44
Patellar Tilt Angle (deg)	24.8

The DICOM image datasets were processed using Mimics Research software (Materialise, Belgium, Version 21.0). Multiplanar segmentations of the femoral, tibial, and patellar bony surfaces, along with the menisci, were performed using the software’s built-in automatic thresholding functions. These initial segmentations were further refined manually on a slice-by-slice basis using advanced editing tools to ensure high anatomical precision. The finalized segmented structures were converted into three-dimensional (3D) surface models and exported as stereolithography (STL) files for further refinement.

For enhancement of model quality, Geomagic Wrap 2021 (Geomagic Corp., United States) was utilized for remeshing and surface smoothing. Advanced techniques, including defeaturing, relaxation, and spike removal, were systematically applied to eliminate artifacts that could potentially compromise simulation accuracy. Contours were identified to construct surface patches and grids, which were fitted to the refined surfaces. The final models were then exported in STEP format for additional processing in SolidWorks 2021 (Dassault Systèmes, France).

In SolidWorks, anatomically matched surface entities for the patellar, femoral, and tibial cartilages, were generated using the software’s offset surface commands, with each structure modeled to a uniform thickness of 2 mm. The assembly of the bone components, cartilage surfaces and menisci were accomplished through the use of origin mate and move duplicate entities commands. Soft tissue structures, including the patellar tendon, MPFL, medial patellotibial ligament (MPTL), and medial patellomeniscal ligament (MPML), were modeled based on anatomical data from Tanaka et al. (2019), Huddleston et al. (2020), employing lofted boss commands.

**FIGURE 1:**
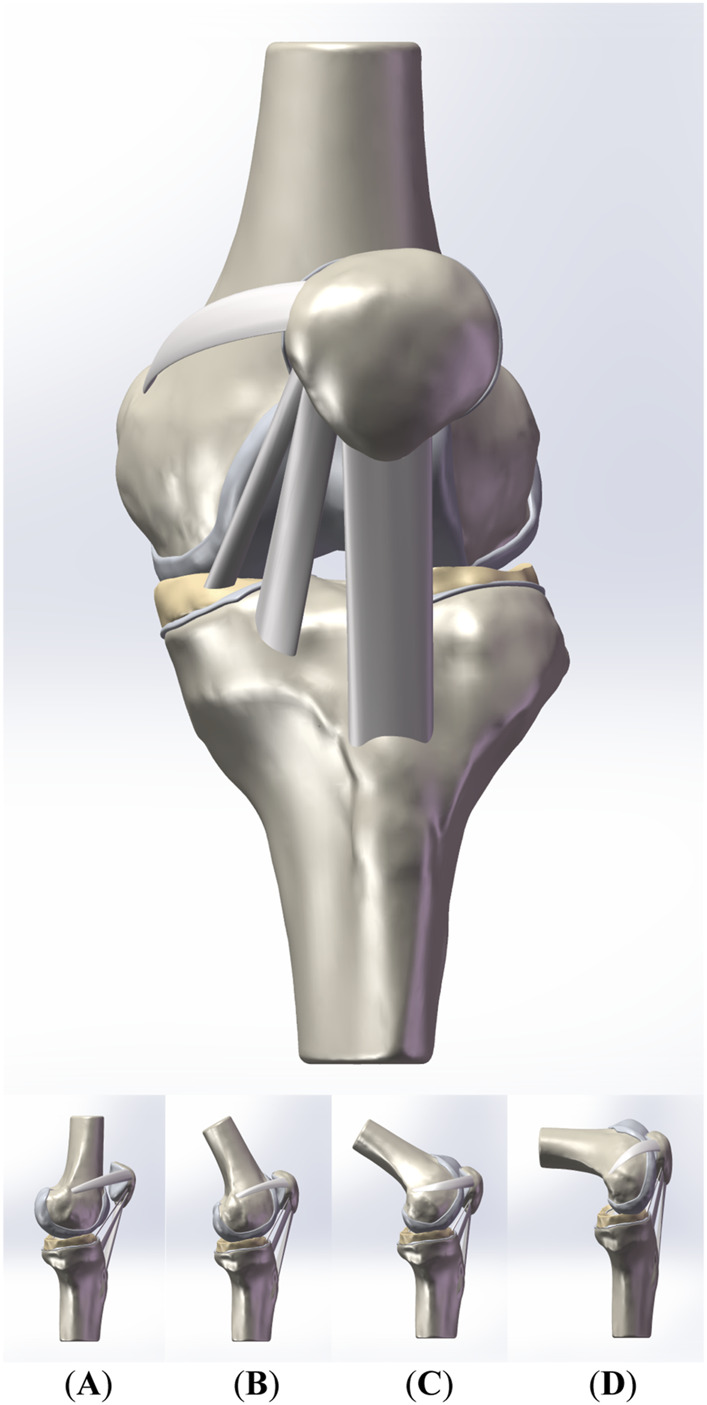
The front view and the side view of the patellofemoral joint model. **(A–D)** are the side view of the models of the PFJ in 0°,30°, 60°, and 90° of knee flexion, respectively.

This comprehensive methodology facilitated the creation of a subject-specific PFJ model that accurately represented abnormal patellar positioning at 0° of knee flexion. From this baseline model, we derived additional models at flexion angles of 30°, 60°, and 90°, guided by the rotational axis studies of Churchill et al. (1998) and the patellofemoral positioning research conducted by Baldwin et al. (2009), as illustrated in Figure 1. Joint motion was modeled based on established kinematic principles, describing knee movement as simultaneous rotations around the true flexion axis—fixed to the femur through the posterior condyle—and the longitudinal rotation axis, aligned with the tibial mechanical axis (Churchill et al., 1998; Baldwin et al., 2009). In SolidWorks, rotational and translational commands were applied to simulate these flexion angles, achieving realistic joint kinematics consistent with widely accepted methodologies (Kaiser et al., 2021; Salvatore et al., 2022). This structured approach ensured precise replication of knee joint motion across various flexion angles and provided a reliable framework for simulating PFJ mechanics.

### 2.2 Material properties, meshing and boundary conditions

In accordance with established biomechanical principles, the components of the PFJ model were defined as isotropic linear elastic materials, with specific material properties outlined in Table 2. The selection of these isotropic materials was informed by previous research (Chen et al., 2023; Smeets et al., 2017; Aykanat et al., 2023), which demonstrated their effectiveness in accurately modeling joint mechanics.

**TABLE 2:** Material properties of various anatomical structures.

Anatomical structures	Material model	Young (MPa)	Poisson ratio	Stiffness (N/mm)
Bone	Isotropic linear elasticity	19,100	0.30	—
Cartilage	Isotropic linear elasticity	15	0.30	—
Meniscus	Isotropic linear elasticity	59	0.49	—
Patellar tendon	Isotropic linear elasticity	225	0.30	—
MPFL, MPTL, MPML	Isotropic linear elasticity	294.6	0.30	—
Lateral retinaculum	Liner tension only spring	—	—	97

Interactions among bone-ligament, bone-cartilage, and cartilage-meniscus interfaces were defined as bonded to ensure stable connections. The PFJ contact was modeled as frictional, with a coefficient of 0.02, as established by Kaiser et al. (2021), to accurately simulate realistic joint behavior under physiological loading conditions.

The model was discretized into tetrahedral elements, with a mesh size of 2-mm for bone tissues and 1-mm for soft tissues, resulting in a total of 542,264 elements and 829,957 nodes. This mesh density exceeds the recommended minimum threshold of 350,000 nodes for biomechanical analyses, thereby enhancing the accuracy of the simulations (Viceconti et al., 2005).

The lateral retinacular was modeled as a spring with a stiffness of 97 N/m, consistent with biomechanical data reported by Merican et al. (Merican et al., 2009). The femur and tibia were constrained in all six degrees of freedom, while the patella remained kinematically unconstrained to allow for realistic joint motion. A force of 175 N was applied to the proximal patella to simulate quadriceps pull (Salvatore et al., 2022; Kheir et al., 2022). Specifically, the quadriceps femoris is divided into five parts: the rectus femoris and vastus intermedius (RF and VI), vastus lateralis lungo (VLL), vastus lateralis obliqus (VLO), vastus medialis longus (VML), vastus medialis obliqus (VMO). Based on the angles of these five parts relative to the femoral shaft and their respective physiological cross - sectional areas, the direction and force distribution details of the five parts are as follows: The RF and VI are oriented at 0° anterior and 0° lateral and undertake 35% of the total force. The VLL is at 0° anterior and 14° lateral, accounting for 33% of the total force. The VLO is positioned at 33° posterior and 35° lateral, representing 9% of the total force. The VML is at 0° anterior and 15° medial, contributing 14% of the total force. The VMO is located at 44° posterior and 47° medial, making up 9% of the total force. This force application was designed to replicate the non - weight bearing and open chain physiological loading conditions experienced during knee flexion.

### 2.3 Model validation and finite element analysis

To validate the FE model of the PFJ, lateral translation tests were conducted at knee flexion angles of 0°, 30°, 60°, and 90°. These simulations assessed lateral patellar stability by applying a lateral displacement force to the patella, shifting it 10 mm laterally while the femur and tibia remained fixed. For each specified knee flexion angle, the force required to achieve this displacement was evaluated. The validation process systematically benchmarked the calculated forces from the FE simulations for the lateral translation test against established values reported in the literature (Amis et al., 2008). The literature we referred to reported the forces required for lateral patellar displacement at different knee flexion angles of 0°, 30°, 60° and 90° were 78.0 ± 8.0 N, 78.0 ± 15.0 N, 102.0 ± 18.0 N and 112.0 ± 21.0 N respectively. A satisfactory correlation between the FE model results and the experimental data was deemed essential for confirming the model’s reliability.

FE analysis was performed using ANSYS Workbench 17 (ANSYS Inc., Canonsburg, PA, United States). To simulate varying degrees of LRR, the stiffness of the lateral retinaculum was incrementally reduced. In models representing MPFL deficiency, the MPFL entity was suppressed to represent a rupture. The resulting mechanical responses, including stress distributions and peak stress, were rigorously analyzed utilizing the post-processing capabilities of ANSYS Workbench.

### 2.4 Statistical methods

For the comparison between partial release and complete release, we paired the data according to the knee flexion angle and the integrity of the medial patellofemoral ligament to eliminate the influence of confounding factors. Then, in GraphPad Prism 9.5 software, we used the Shapiro-Wilk test to evaluate the normality of the data. When the paired differences followed a normal distribution, a paired sample t-test for the means was conducted; otherwise, a Wilcoxon signed-rank sum test was performed. For the comparison among different knee flexion angles, we carried out an analysis of variance for the data from a randomized block design and Tukey’s multiple comparisons test. Statistical significance was defined as P < 0.05.

## 3 Results

### 3.1 Model validation

**FIGURE 2:**
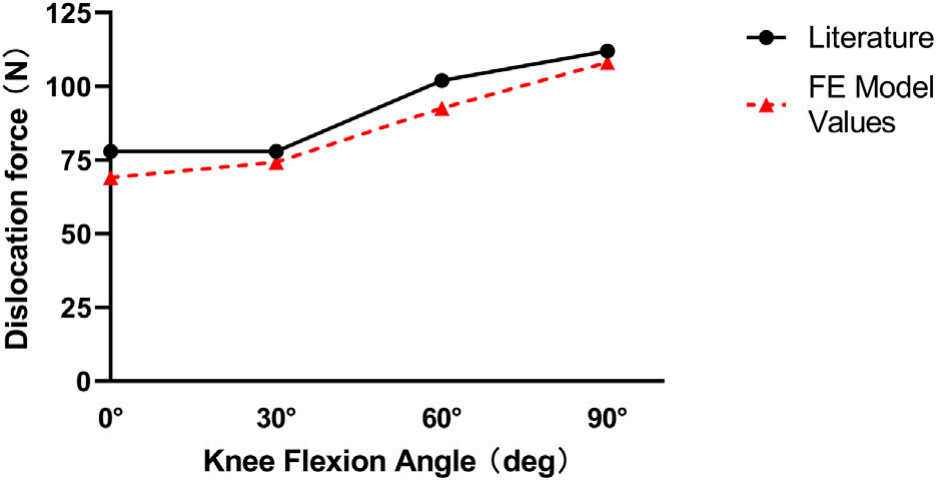
Graphic model validation between the values of the force necessary to laterally dislocate the patella by 10 mm calculated in our model and the reported mean values of the literature.

The FE model accurately predicted the forces required for lateral patellar displacement across the knee flexion angles studied. Specifically, the forces obtained were 69.1 N at 0°, 74.4 N at 30°, 92.6 N at 60°, and 108.2 N at 90° of flexion. These values fell within the reported standard deviation range and were in close agreement with experimental data reported in literature, as illustrated in Figure 2. The Pearson correlation coefficient between our model values and the literature values is r = 0.9845, indicating a very strong positive linear relationship. Additionally, the p - value associated with this correlation is p = 0.0155. This consistency between the model’s predictions and empirical data further supports the validity of the FE model in replicating the mechanical behavior of the patellofemoral joint.

### 3.2 Effect of knee flexion angle on the PFJ

**FIGURE 3:**
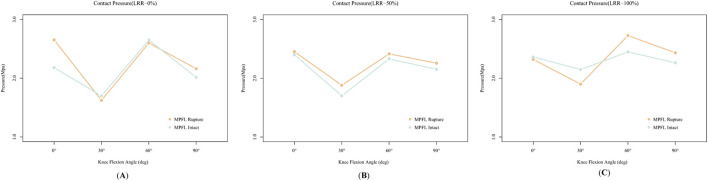
The effects of medial patellofemoral ligament (MPFL) rupture or not on patellofemoral joint (PFJ) contact pressure across different knee flexion angles. **(A–C)** are under intact, partially released, and completely released lateral retinacular, respectively.

**FIGURE 4:**
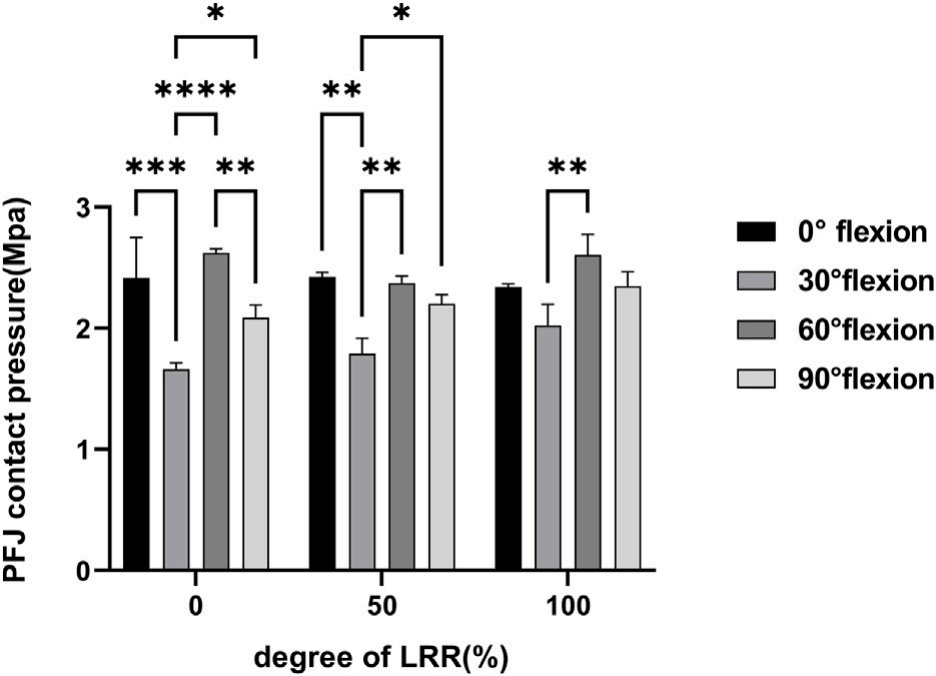
Multiple comparisons of the patellofemoral joint pressure among different knee flexion angles.

The finite element (FE) analysis revealed significant variability in patellofemoral joint (PFJ) contact pressure across a range of knee flexion angles, as illustrated in Figure 3. Specifically, throughout 0°–90° of knee flexion, PFJ contact pressures were notably elevated at 0° and 60°, while comparatively lower at 30° and 90°. The results of pairwise comparisons among different knee flexion angles are shown in Figure 4. When the lateral retinaculum is intact, the differences among various angles are the most obvious. There are statistically significant differences between 30° and other knee flexion angles (the p-values compared with 0°, 60° and 90° were 0.0007, <0.0001 and 0.0398, respectively). Moreover, there is also a significant difference between 60° and 90°, with p-value was 0.0100. When the lateral retinaculum is partially released, there are still statistically significant differences between 30° and other knee flexion angles (the p-values compared with 0°, 60° and 90° were 0.0029, 0.0056 and 0.0472, respectively). When the lateral retinaculum is completely released, there is a difference only between 30° and 60°, with p-value was 0.0055.

Under the combined influence of these malalignment conditions, the inferior pole of the patella impinges on the lateral femoral condyle, leading to an initial peak in PFJ contact pressure at 0° of knee flexion. As the knee flexes from the neutral 0° position, the contact pressure progressively decreases, reaching its minimum when the patella is fully seated within the trochlear groove at approximately 30° of flexion. Beyond this angle, the PFJ contact pressure begins to increase steadily, peaking around 60° of flexion. This secondary peak is attributed to the descent of the patella along the trochlear groove, coupled with the posterior force exerted by the quadriceps, which shifts the center of pressure toward the mid-upper region of the patellar crest. At 90° of knee flexion, the contact forces on the PFJ diminish due to femoral condyle structure and partial balancing of medial and lateral quadriceps forces, resulting in a reduction in peak PFJ contact pressure.

This biomechanical pattern was consistent across both the MPFL-intact and MPFL-deficient models, indicating a persistent alteration in joint mechanics, irrespective of MPFL integrity or the extent of LRR. Consequently, these findings suggest that abnormal patellar positioning significantly affects the distribution of PFJ contact pressure.

### 3.3 Effect of LRR on the PFJ

**FIGURE 5:**
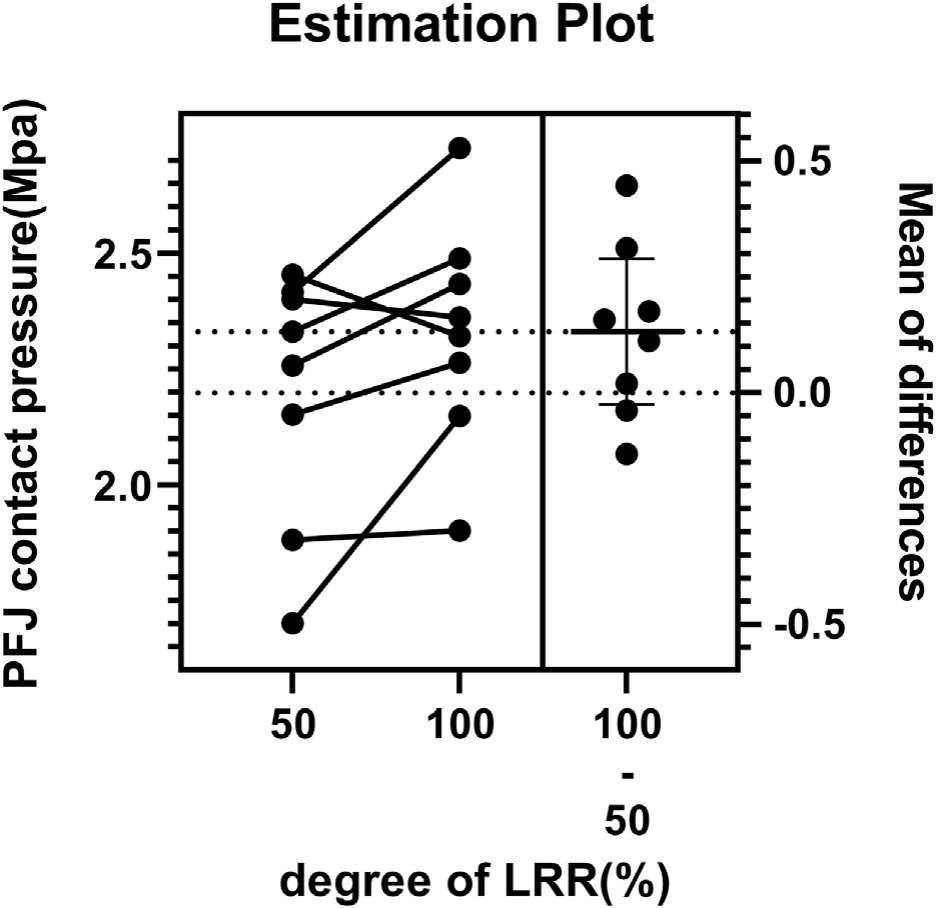
Estimation plot of the paired comparison of patellofemoral joint pressure between partial LRR and complete LRR.

Despite the absence of a statistically significant difference in patellofemoral joint (PFJ) contact pressure between partial release and complete release (P = 0.0874), on the whole, upon complete release, the PFJ contact pressure demonstrates a propensity to rise, as illustrated in Figure 5.

**FIGURE 6:**
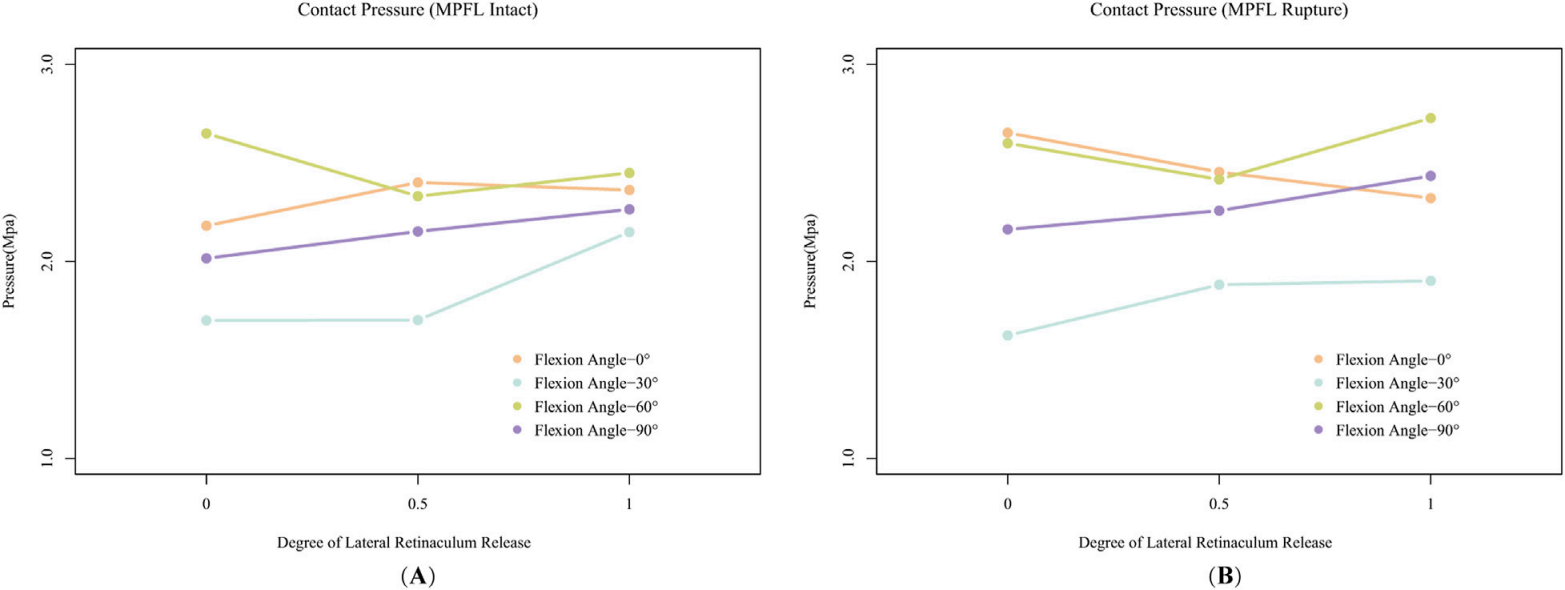
The effects of varying degrees of Lateral Retinacular Release (LRR) on patellofemoral joint (PFJ) contact pressure. **(A)** is the result of the model with an intact medial patellofemoral ligament (MPFL), while **(B)** is the result of the model simulating a ruptured MPFL.

In the FE model with an intact MPFL, varying degrees of LRR exerted differential impacts on PFJ pressures across different angles of knee flexion, as illustrated in Figure 6A.

At 0° of knee flexion, a partial release (50%) of the lateral retinaculum resulted in increased PFJ pressure. Although complete release (100%) slightly reduced the PFJ pressure at the same flexion angle compared to the partial release condition, the pressure remained elevated relative to the scenario with no release.

At 30° of knee flexion, partial LLR did not alter PFJ pressures; however, complete release increased PFJ pressures to levels comparable to those observed at 60° and 90° of flexion.

At 60° of knee flexion, partial release sharply decreased PFJ pressure, while complete release caused a slight increase; however, these pressures were still lower than those recorded without any release.

At 90° of knee flexion, PFJ pressures progressively increased with greater degrees of LRR.

In the model simulating a ruptured MPFL (Figure 6B), the response of PFJ pressure to LRR exhibited notable differences. At 0° of knee flexion, PFJ pressure decreased with increasing degrees of release. At 30°, partial release resulted in increased PFJ pressure, whereas complete release did not substantially alter the pressure. At 60° of flexion, partial release reduced PFJ pressure; however, complete release resulted in a sharp rebound, surpassing the pressure levels observed without any release. The pressure trend at 90° of flexion mirrored that of the intact MPFL model, with pressures increasing progressively in relation to the extent of release.

**FIGURE 7:**
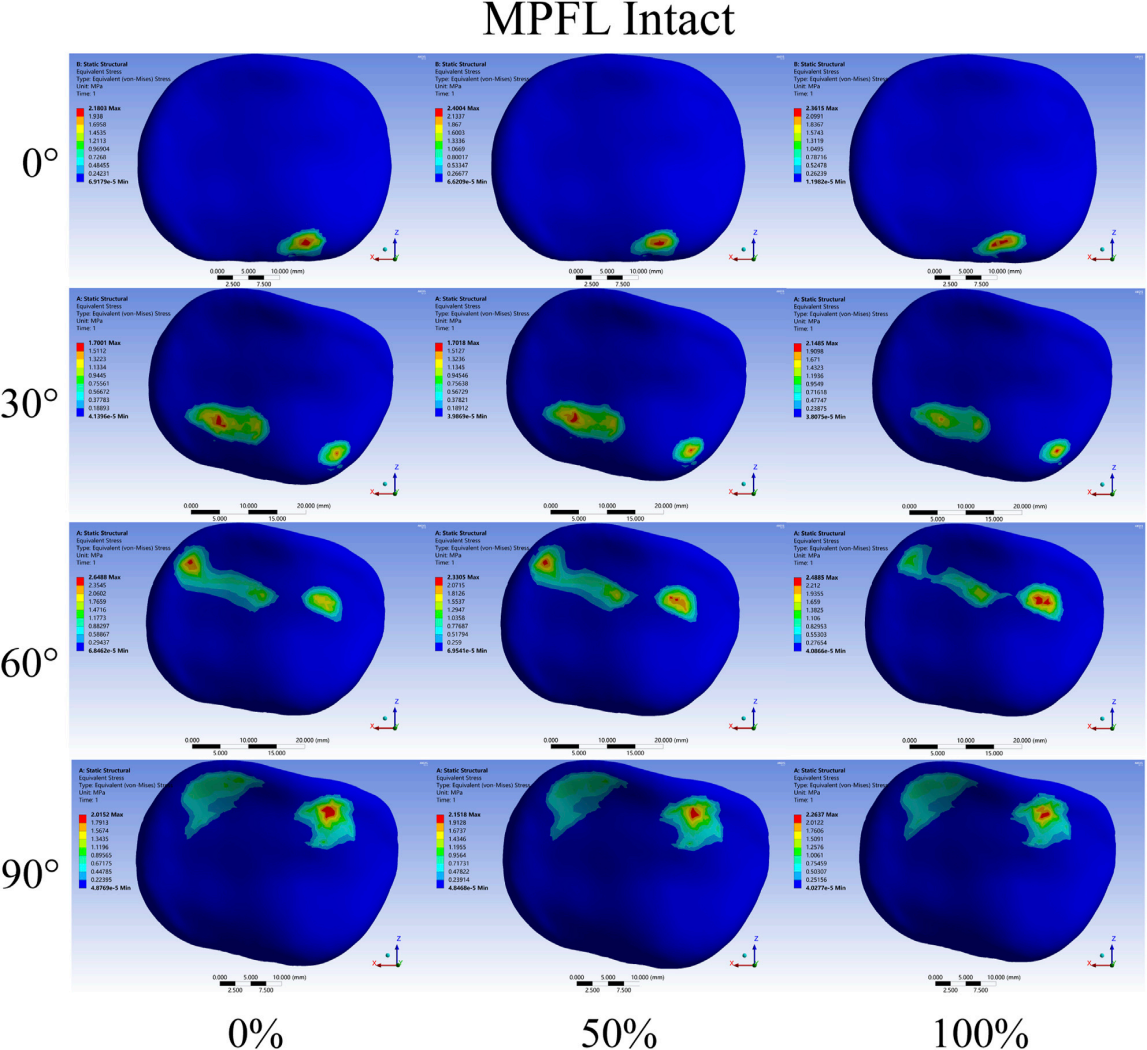
Contact pressure distribution of patellar cartilage when the medial patellofemoral ligament (MPFL) is intact. The extent of release increasing from left to right. The knee flexion angles from top to bottom are 0°,30°, 60°, and 90°.

**FIGURE 8:**
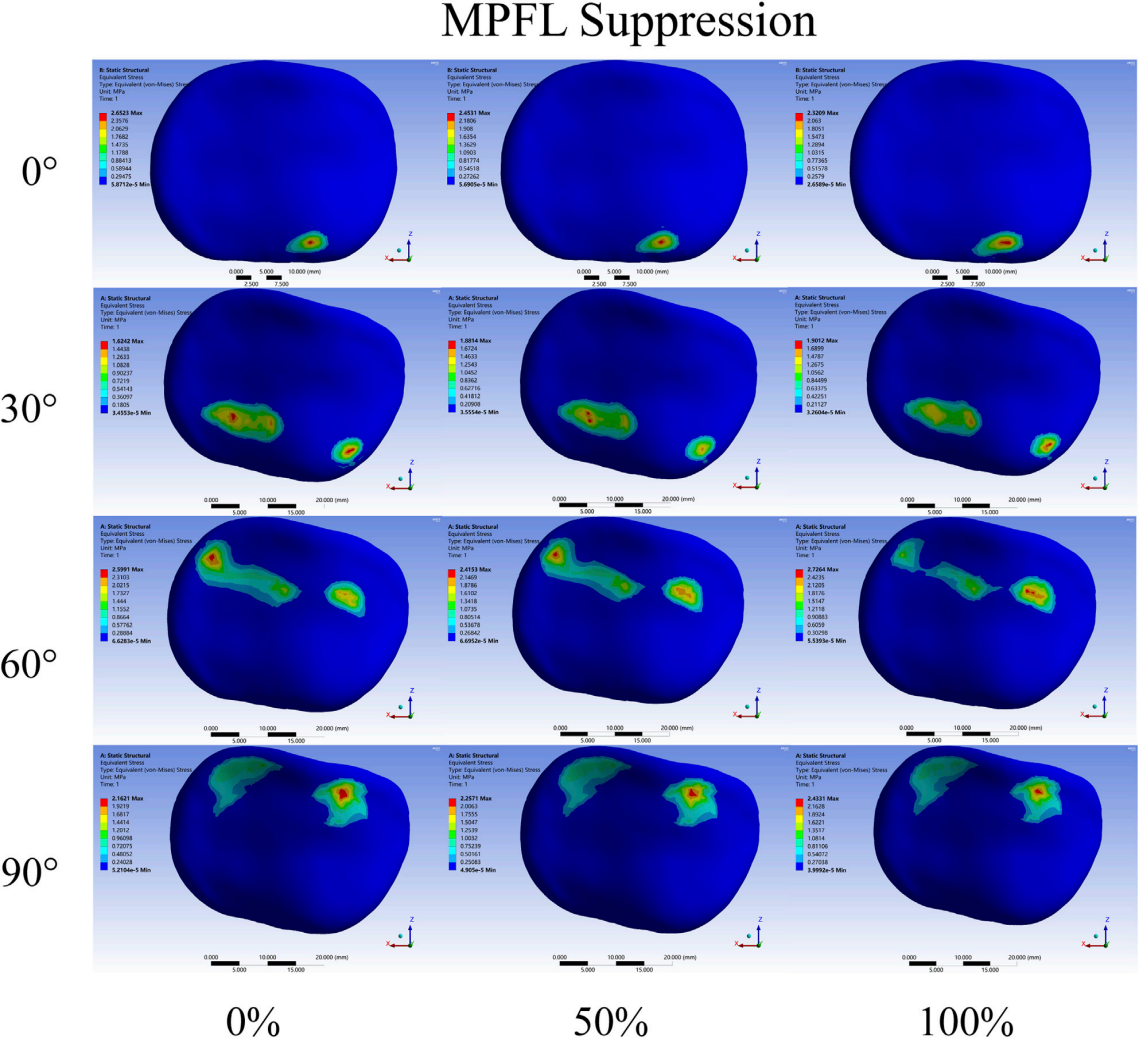
Contact pressure distribution of patellar cartilage in rupture of the medial patellofemoral ligament (MPFL). The extent of release increasing from left to right. The knee flexion angles from top to bottom are 0°,30°, 60°, and 90°.

Regarding the distribution of PFJ pressure (Figures 7, 8), at 0° of knee flexion, the pressure center consistently remained medial to the patellar crest at the inferior pole of the patella, demonstrating no significant change with either partial or complete release. At 30° and 60° of flexion, especially complete release led to a decrease in lateral PFJ pressure and an increase in medial pressure, resulting in a shift of the pressure center from lateral to medial. At 90° of knee flexion, the pressure center was consistently located on the medial patellar articular surface, with the degree of release (partial or complete) affecting only the peak pressure, rather than the position of the pressure center.

### 3.4 Effect of MPFL rupture on the PFJ

In the models with an intact MPFL, the mean PFJ contact pressure was measured at 2.2189 ± 0.2607 MPa across various degrees of LRR and knee flexion angles. In contrast, the MPFL-ruptured group exhibited a slightly higher mean PFJ contact pressure of 2.2945 ± 0.3127 MPA, with a statistically significant difference observed between the two groups (P = 0.045).

When the lateral retinaculum was intact, MPFL rupture resulted in a significant increase in PFJ pressure at 0° of knee flexion, with minimal changes noted at other flexion angles. Following a partial LRR, the MPFL rupture caused a slight increase in PFJ pressure across all knee flexion angles. Conversely, after a complete LRR, the MPFL rupture was associated with a decrease in PFJ pressure at 30° of flexion, while increases were recorded at 60° and 90° flexion of flexion.

## 4 Discussion

In contrast to prior biomechanical studies that primarily focus on medial and lateral patellar displacement (Kheir et al., 2022; Cancienne et al., 2019), the current research presents novel insights into the biomechanical effects of partial and complete LRR on PFJ contact pressures across clinically relevant knee flexion angles in patients with type A-B trochlear dysplasia, patella alta, and elevated TT-TG values. These findings significantly advance the understanding of PFJ mechanics in patients predisposed to lateral patellar instability, highlighting the importance of informed decision-making in surgical planning for this patient population.

To strengthen the validity of these findings, we also conducted lateral translation simulations at key knee flexion angles (0°, 30°, 60°, and 90°), closely replicating the conditions of A.A. Amis’s cadaveric study, with good agreement observed between our results and the experimental data (Amis et al., 2008). Our model accurately reflects PFJ mechanics by incorporating essential stabilizing structures, including—the patellar tendon, quadriceps muscle, medial patellofemoral ligament (MPFL), medial patellotibial ligament (MPTL), medial patellomeniscal ligament (MPML), and the lateral retinaculum—which collectively account for over 90% of PFJ stability during flexion and extension (Philippot et al., 2012).

Additionally, the lateral retinaculum can be anatomically divided into the iliotibial band–patella (ITB-P) fibers, lateral patellofemoral ligament, and lateral patellomeniscal ligament. Notably, the ITB-P fibers are the thickest component, exhibiting a stiffness of 97 N/m, with an average strength that is three to six times greater than that of the lateral patellofemoral and patellomeniscal ligaments (Merican et al., 2009; Merican and Amis, 2008). Thus, it plays a crucial role in maintaining the normal trajectory of patellar movement. Our simulation closely mirrors clinical practice, as it considers the entire lateral retinaculum rather than focusing solely on individual ligaments, which aligns with the comprehensive evaluations typically performed during preoperative planning and surgical procedures. Furthermore, understanding the structure and role of the lateral retinaculum provides context for interpreting the pressure variations observed in our study.

The biomechanical insights obtained from this study have significant implications for surgical practice.

Firstly, partial release of the lateral retinaculum may be more appropriate. Contrary to initial expectations, complete LRR does not consistently reduce PFJ contact pressure. The paired sample t-test results showed that there was no significant difference in PFJ contact pressure between partial release and complete release. What’s more, at 30°, 60°, and 90° of knee flexion, complete LRR actually results in higher contact pressure than partial release. This may due to the PFJ contact pressure and center position are the balanced results of the bony constraints and essential soft tissue structures (Pohlig et al., 2021). After complete LRR, losing the antagonism of the lateral retinaculum, the peak pressure at the new contact point may be higher.

Specifically, compared to partial release, complete release induces a more significant shift in the pressure center from the lateral to the medial side, which can lead to visibly higher pressures at the new contact points. This shift may cause increased stress on the medial cartilage and underlying bone, potentially contributing to medial patellofemoral pain and degeneration. However, partial release could better relocating the pressure center of the PFJ from the lateral to the medial aspect without substantially increasing medial compartment loading, thereby reducing the risk of the aforementioned complications.

Moreover, partial LRR reduces PFJ contact pressure at the critical angle of 60° knee flexion, where PFJ pressure is typically high without release. Besides, it won't elevate the PFJ pressure when MPFL was intact at 30° knee flexion like what happens in the case of complete release. This is especially beneficial for symptomatic relief in these patients, as it addresses a mechanically demanding phase of knee motion.

Of note, expanding the degree of LRR may amplify the impact of MPFL rupture on PFJ contact pressure. When the lateral retinaculum is intact, the MPFL rupture will lead to the increase of PFJ contact pressure only in the extension position; However, in the case of MPFL rupture, the PFJ contact pressure across all knee flexion angles would slightly increase after partial LRR; An especially sharp increase of PFJ contact pressure at 60° and 90° of knee flexion was observed after complete LRR. Therefore, it is better to perform LRR surgery on the premise of ensuring the integrity of the MPFL. Alternatively, the LRR surgery should be performed simultaneously with the MPFL repair or reconstruction surgery (Gallagher et al., 2022; Wang et al., 2021b).

Additionally, the lateral stabilizing structures, contribute uniquely to PFJ stability and pressure distribution compared to the MPFL. Our analysis across knee flexion angles of 0°, 30°, 60°, and 90° suggests that while the MPFL plays a critical role in limiting lateral patellar displacement, its effect on pressure changes within the PFJ is comparatively less significant. This finding may explain why patients with recurrent patellar dislocation frequently report feelings of instability and apprehension post-injury, yet experience relatively less pain within or around the PFJ.

Secondly, the analysis derived from our FE model indicates that relatively high PFJ contact pressures at 0° of knee flexion, particularly when the inferior pole of the patella contacted the lateral femoral condyle, which is an outcome likely influenced by unique anatomical characteristics. Partial or complete LRR at 0° knee flexion may weaken the antagonistic horizontal forces, leading to further increased PFJ contact pressure with the MPFL intact. Furthermore, our FE results also demonstrated pressures are lowest at 30°of knee flexion and highest at 60°, which is consistent with results reported by Salvatore et al. (2022). This pattern suggests that such malalignment, including mild trochlear dysplasia, patella alta, and an elevated TT-TG distance (type A-B femoral trochlear dysplasia, I-S ratio ≈1.4, TT-TG value ≈20 mm), can disrupt normal PFJ biomechanics, intensifying joint stresses and contributing to the distinct pressure distributions observed in this study.

Building on our findings regarding knee flexion angles with intact lateral retinaculum, our research also showed that the effect of LRR on PFJ contact pressure is distinct under different knee flexion angles. Exactly at 60° of knee flexion and 0° of knee flexion after MPFL rupture, the PFJ contact pressure decreased visibly after partial release of the lateral retinaculum. Under the other knee flexion angles, the PFJ contact pressure increased in varying degrees.

Hence, the importance of carefully monitoring patellar alignment at critical flexion angles (0°, 30°, 60°, and 90°) during surgery cannot be overstated. Evaluating contact positions and pressure distribution at these critical angles can help determine whether LRR is required and if the degree of release is appropriate. Such intraoperative evaluations can also help avoid potential complications associated with excessive LRR, especially in patients undergoing MPFL reconstruction (Kheir et al., 2022).

Furthermore, during these minimally invasive arthroscopic procedures, adjunct intraoperative imaging—including dynamic fluoroscopic imaging for continuous assessment of patellar tracking and alignment, and ultrasound for detailed soft tissue visualization—can enhance real-time evaluation. Dynamic fluoroscopy allows continuous monitoring of patellar tracking across flexion angles, providing immediate feedback on bony alignment and implant positioning, while ultrasound offers insights into ligament tension and soft tissue dynamics without additional radiation exposure. Together, these imaging modalities can enhance arthroscopic visualization, providing a comprehensive intraoperative assessment of both osseous and soft tissue structures, which is essential for achieving optimal patellar alignment and stability, in turn enhancing surgical outcomes in these patients.

## 5 Future scope

This study lays the groundwork for personalized surgical planning through patient-specific anatomical and biomechanical models. Future research could expand on this by integrating high-precision CT and MRI data to create a 3D model database that simulates surgical interventions. This approach would allow for more accurate predictions of surgical outcomes, helping to standardize treatment protocols, reduce complications, and shorten the learning curve for young surgeons. In order to refine the accuracy of surgical predictions and achieve real-world applications, future research could also expand on this by integrating temporal effects and viscoelastic properties to better capture transient responses during movement and more accurately reproducing stress and deformation patterns under dynamic conditions (Xu et al., 2024; Xu et al., 2023). In addition, clinical trials are essential to validate the findings of this study. Prospective clinical trials comparing partial LRR and complete LRR in a large number of patients can directly assess the effectiveness and safety of the two procedures in terms of clinical outcomes, such as pain relief, functional improvement, and long - term stability.

## 6 Limitation

This study has several limitations. Firstly, we used a single patient model for the finite - element analysis. Although the case selected in our current study represents an important clinical scenario in orthopedic practice, future studies should increase the number of cases to enhance the generalizability of the findings. Secondly, while the assumption of isotropic and linear elasticity for ligaments is commonly used in biomechanical studies and sufficient for modeling typical loading conditions, future research should incorporate more advanced material models to fully capture the more profound biomechanical behavior of ligaments. Thirdly, the static nature of our simulation limits its direct applicability to dynamic activities like walking or running. However, this study provides valuable insights into patellofemoral joint mechanics under controlled conditions, serving as an important step toward understanding PFJ stability and guiding future research in dynamic simulations.

## 7 Conclusion

Our study provides new theoretical basis for the expected outcomes of varying degrees of LRR, which helps clinicians better conduct preoperative planning, especially in avoiding over - aggressive LRR procedures which may not yield improved outcomes. Partial LRR offers significant biomechanical advantages over complete release. It effectively reduces PFJ contact pressure and minimizes the risks associated with complete LRR, such as increased medial compartment pressure and worsened MPFL rupture. Given that complete LRR does not provide additional benefits and can introduce risks, partial LRR should be considered the superior approach for managing PFJ instability in this patient population. For future research, dynamic simulations and large - scale clinical trials are promising directions to further validate and expand on our findings.

## Data Availability

The original contributions presented in the study are included in the article/supplementary material, further inquiries can be directed to the corresponding author.
